# Evidence on Digital Mental Health Interventions for Adolescents and Young People: Systematic Overview

**DOI:** 10.2196/25847

**Published:** 2021-04-29

**Authors:** Susanna Lehtimaki, Jana Martic, Brian Wahl, Katherine T Foster, Nina Schwalbe

**Affiliations:** 1 Spark Street Advisors New York, NY United States; 2 Department of International Health Johns Hopkins Bloomberg School of Public Health Baltimore, MD United States; 3 Department of Psychology University of Washington Seattle, WA United States; 4 Department of Global Health University of Washington Seattle, WA United States; 5 Heilbrunn Department of Population and Family Health Columbia Mailman School of Public Health New York, NY United States; 6 United Nations University International Institute for Global Health Kuala Lumpur Malaysia

**Keywords:** digital health, adolescent health, young people, mental health, digital technologies

## Abstract

**Background:**

An estimated 1 in 5 adolescents experience a mental health disorder each year; yet because of barriers to accessing and seeking care, most remain undiagnosed and untreated. Furthermore, the early emergence of psychopathology contributes to a lifelong course of challenges across a broad set of functional domains, so addressing this early in the life course is essential. With increasing digital connectivity, including in low- and middle-income countries, digital health technologies are considered promising for addressing mental health among adolescents and young people. In recent years, a growing number of digital health interventions, including more than 2 million web-based mental health apps, have been developed to address a range of mental health issues.

**Objective:**

This review aims to synthesize the current evidence on digital health interventions targeting adolescents and young people with mental health conditions, aged between 10-24 years, with a focus on effectiveness, cost-effectiveness, and generalizability to low-resource settings (eg, low- and middle-income countries).

**Methods:**

We searched MEDLINE, PubMed, PsycINFO, and Cochrane databases between January 2010 and June 2020 for systematic reviews and meta-analyses on digital mental health interventions targeting adolescents and young people aged between 10-24 years. Two authors independently screened the studies, extracted data, and assessed the quality of the reviews.

**Results:**

In this systematic overview, we included 18 systematic reviews and meta-analyses. We found evidence on the effectiveness of computerized cognitive behavioral therapy on anxiety and depression, whereas the effectiveness of other digital mental health interventions remains inconclusive. Interventions with an in-person element with a professional, peer, or parent were associated with greater effectiveness, adherence, and lower dropout than fully automatized or self-administered interventions. Despite the proposed utility of digital interventions for increasing accessibility of treatment across settings, no study has reported sample-specific metrics of social context (eg, socioeconomic background) or focused on low-resource settings.

**Conclusions:**

Although digital interventions for mental health can be effective for both supplementing and supplanting traditional mental health treatment, only a small proportion of existing digital platforms are evidence based. Furthermore, their cost-effectiveness and effectiveness, including in low- and middle-income countries, have been understudied. Widespread adoption and scale-up of digital mental health interventions, especially in settings with limited resources for health, will require more rigorous and consistent demonstrations of effectiveness and cost-effectiveness vis-à-vis the type of service provided, target population, and the current standard of care.

## Introduction

### Background

Mental health issues remain underdiagnosed and undertreated among adolescents and young people (aged 10-24 years) [1]. Ignored by many health and social services and policies worldwide [2], adolescents and young people are particularly vulnerable to many conditions affecting mental health. Nearly 50% of mental health disorders begin by the age of 14 years, and 75% of mental health disorders begin by the age of 24 years [3]; an estimated 1 in 5 adolescents experience a mental health disorder each year [4]. The emergence of symptom sequelae, even below the diagnostic threshold, signals an increased vulnerability to life course–persistent mental health problems and consequences if not addressed early. Among men and women aged between 15-19 years, suicide, which is more common among young people than adults [5], is one of the top 3 causes of death worldwide, and depression is among the leading causes of disability for those aged between 10-19 years [6].

At the same time, young people are growing up in the digital world and accessing the internet at increasingly younger ages [7]. As the most connected age group in the population, more than 70% of young people aged between 15-24 years are “online” [8]. Although there are income-based and geographical disparities in digital access, 43% of people in low- and middle-income countries use the internet, and even in low-income countries, 72% of people have access to mobile phones, and 16% of people have access to the internet [9].

Although there are clearly some negative effects of technology on this age group, including behavioral addiction, cyber-bullying, depression, sexual exploitation, and abuse [10-12], the use of digitally enabled technology is considered a promising platform for preventing morbidity and enhancing well-being and quality of life [13]. Critically, digital technologies may offer especially critical support for adolescents and young people in low-resource settings where barriers to care may be numerous and insurmountable.

Given the increasing number of adolescents and young people using digital technologies, digital mental health interventions are considered to have the specific potential to support mental health and well-being in this group [14,15]. Specifically, digital technology could provide opportunities to access mental health services and information while also increasing patient empowerment, participation [16], and help-seeking and helping to overcome the stigma that is often linked to mental health services [17]. With more than 2 million mental health apps already available, including 40,000 classified as medical [18], the demand for this innovation is evident. However, the plethora of these apps may have outpaced the development of a correspondingly large evidence base on their effectiveness.

### Objectives

A number of systematic reviews and meta-analyses have been conducted over the past 10 years on the use of digital technology to enhance mental health among adolescents and young people. A higher-level synthesis of information across these meta-analyses and reviews is needed to identify whether there is converging evidence for their effectiveness and to assess systematic issues with research in this area. Consequently, this systematic overview provides a high-level synthesis of the current evidence on the effectiveness of digital health interventions targeting adolescents and young people (ie, aged 10-24 years as defined by the World Health Organization and others [19,20]; Textbox 1) with diagnosed or self-reported mental health conditions, including affective, behavioral, and trauma-related conditions (eg, anxiety, depression, psychological distress, eating disorders, and posttraumatic stress disorder). Furthermore, it aims to characterize the factors, including digital platforms and design elements used, that contribute to the effectiveness. Finally, it aims to describe the extent to which there is evidence of the economic benefits of such interventions and determine the extent to which previous research in this area may generalize to low-resource settings, including low- and middle-income countries.

The research questions are as follows:

- In adolescents and young people aged between 10 and 24 years, to what extent are digital health interventions effective in addressing mental health conditions, compared with standard face-to-face treatment, placebo, or no treatment?
- What factors contribute to effectiveness (ie, what makes effective interventions effective)?
- To what extent is there evidence on cost-effectiveness?
- To what extent are the findings generalizable to adolescents and young people from a range of settings, including low- and middle-income countries?

**Definitions of key terms.**

Adolescents and young people

- According to the World Health Organization, adolescents are individuals aged 10-19 years, and young people are individuals aged 10-24 years [19]

Mental health conditions, mental disorders

- Mental health problems with different symptoms, characterized by a combination of abnormal thoughts, perceptions, emotions, behavior, and relationships with others [21,22]

Digital mental health intervention

- Information, support, and therapy for mental health conditions delivered through an electronic medium with the aim of treating, alleviating, or managing symptoms [23,24]

Cognitive behavioral therapy (CBT), computerized CBT (cCBT)

- A form of psychological treatment to identify maladaptive patterns of thinking, emotional response, or behavior and substituting them with desirable patterns [25]. cCBT refers to computerized implementation of CBT

Effectiveness, effect

- The ability of an intervention to produce intended outcomes, estimated by comparing the intervention with no intervention (ie, better than nonactive control) and/or an existing evidence-based intervention (ie, no difference from active control) [26]

Active control

- A comparison group receiving standard treatment, including face-to-face therapy, alternative therapy, or materials [26]

Nonactive control

- A comparison group not receiving or performing any activity. These may include placebo treatment, no treatment, or assigned to a waitlist to receive intervention after completion of the trial [26]

## Methods

### Search Strategy and Selection Criteria

The review was conducted using a predefined protocol. We conducted an electronic review of the literature from the MEDLINE, PubMed, PsycINFO, and Cochrane databases. The review was limited to peer-reviewed articles published in English between January 1, 2011, and July 6, 2020. We used a combination of keywords: (“digital,” “mHealth,” “eHealth,” “web-based,” “internet-based,” “mobile phone,” “text message,” “SMS,” “artificial intelligence”) AND (“adolescen*,” “youth,” “young,” “child,” “student”) AND (“mental health,” “wellbeing”). Our search was limited to overview types of studies, such as meta-analyses and systematic reviews.

Identified references were screened independently by 2 reviewers (SL and JM) by conducting an abstract and title search with the following inclusion criteria, following a predefined PICOS (Population, Intervention, Comparator, Outcome, Setting) framework:

- Population: Adolescents and young people, defined as primarily aged between 10 and 24 years (or if older participants were included, the mean age was <25 years), with a mental health condition, including anxiety, affective, and behavioral conditions (diagnosed and self-reported)
- Intervention: Consumer-facing, partially or fully self-administered, mental health intervention delivered through a digital platform (eg, web-based, computer, or mobile phone)
- Comparator: Active (ie, standard nondigital care and alternative materials) or passive control (ie, placebo and no treatment)
- Outcome: Mental health improvement as reported by studies (ie, diagnosed or self-reported mental health conditions, including affective, behavioral, and trauma-related conditions)
- Setting: Nonclinical, nonfacility-based setting in any country

Potentially relevant studies identified through the screening process were assessed independently for final inclusion by 2 reviewers (SL and JM) after being acquired in full text. References were excluded if they were not exclusive to this age group; were delivered at the health care facility (eg, telemedicine by clinicians); targeted adolescents and young people with chronic diseases, such as HIV, diabetes, or cancer; targeted adolescents and young people with mental and behavioral disorders because of psychoactive substance use; or were primarily addressing parenting skills or targeting parents. Study protocols and nonpeer-reviewed papers were excluded.

### Data Extraction and Quality Assessment

In total, 2 reviewers (SL and JM) independently extracted information from the studies, building a matrix including data on participants (age and other available background characteristics), interventions, mental health issues addressed, setting (eg, delivery platforms and countries), and key findings in terms of clinical effectiveness. The reviewers also assessed the quality of the articles by using the AMSTAR 2 (A Measurement Tool to Assess Systematic Reviews) [27] tool, which is a validated tool to analyze the quality of systematic reviews and meta-analyses with ratings from high to critically low. The guidance document of the tool [27] was thoroughly followed. Any disagreement in either of these actions was resolved through discussion.

### Data Synthesis

We synthesized evidence from the articles describing the effectiveness of digital mental health interventions against clinical outcomes, therapy used, and digital platform deployed as well as reviewed factors associated with effectiveness, sustainability of outcomes, completion, and adherence. Finally, we reviewed and synthesized the extent to which there was evidence on the cost-effectiveness and the potential generalizability of the findings to low- and middle-income countries. Given the high heterogeneity of the studies, we did not conduct a statistical analysis.

## Results

### Overview

**Figure 1:**
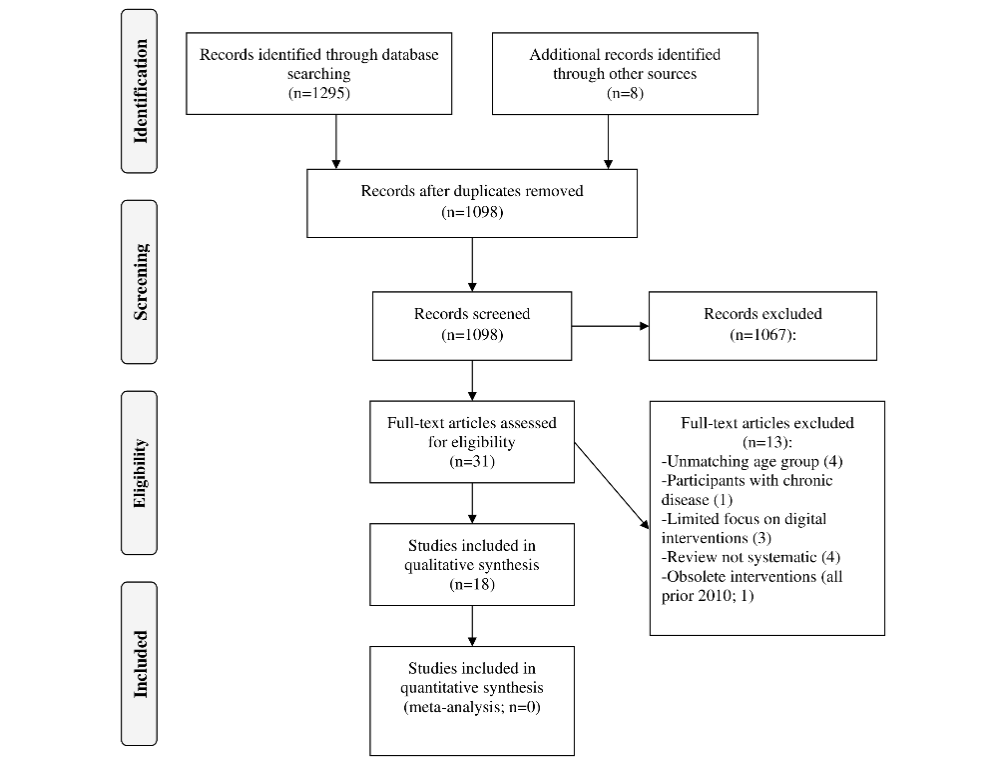
PRISMA (Preferred Reporting Items for Systematic Reviews and Meta-Analyses) flowchart.

The initial search yielded 1295 articles. After excluding duplicate references, the number of articles was reduced to 1098. The search strategy was complemented by a manual search of reference lists of key articles, which yielded an additional 8 articles for eligibility assessment (Figure 1).

After title screening, we conducted full-text appraisal and excluded articles that did not meet the inclusion criteria. A total of 18 articles were finally included (Table 1).

**Table 1:** Study characteristics.

Author	Primary studies	Intervention, digital platform	Outcome	Comparison interventions	Total sample	Participants age (range and mean, if available)	Geographical coverage
Barnes et al (2018) [28]	Systematic review of 2 RCTs^a^ and 3 NRSs^b^ on 3 games	Video games	Anxiety	Nonactive (alternative nontherapeutic video game)	410	Up to 19 years	China, Hong Kong, the Netherlands, and the United Kingdom
Bry et al (2018) [29]	Systematic review and content analysis of 121 apps	Mobile apps	Anxiety	No control group	Not reported	Not defined (children and adolescents)	Not defined
Grist et al (2017) [30]	Systematic review of 23 NRTs^c^ and 1 RCT on 15 apps	Mobile apps	Mental health, well-being, anxiety, depression, suicide, obsessive-compulsive disorder, and eating disorders	Active (nondigital intervention; nb. Only one RCT included in the review)	1054	Up to 18 years	Australia, Canada, Denmark, Ireland, the Netherlands, and the United States
Hollis et al (2017) [24]	Systematic review of 30 RCTs; meta-review of 21 articles on 147 interventions	Internet-based interventions, mobile apps, and eHealth	Anxiety, depression, attention deficit hyperactivity disorder, autism spectrum disorder, psychosis, eating disorders, and posttraumatic stress disorder	Mixed nonactive (waitlist, no intervention) and active (attention control group, limited intervention)	5333	Up to 25 years	Australia, China, the Netherlands, New Zealand, Norway, Israel, Sweden, Switzerland, the United Kingdom, and the United States
Davies et al (2014) [31]	Systematic review of 17 RCTs and meta-analysis of 14 RCTs	Computer-delivered or web-based interventions	Anxiety, depression, psychological distress, and stress	Mixed nonactive (no treatment, waitlist) and active (alternative materials)	1480	17-51 years; mean 22.6 years	Australia, Canada, Norway, Spain, the United Kingdom, and the United States
Farrer et al (2013) [32]	Systematic review of 26 RCTs and 1 randomized trial	Internet-based, audio, virtual reality, and computer programs	Anxiety and depression	Mixed nonactive (no intervention, waitlist) and active (attention control group)	Not reported	18-25 years	Australia, Belgium, China, Italy, the Netherlands, Spain, the United Kingdom, and the United States
Valimaki et al (2017) [33]	Systematic review of 22 RCTs and meta-analysis of 15 RCTs	Internet-based interventions	Depression, anxiety, and stress	Mixed nonactive and active (not specified)	4979	10-24 years	Australia, Canada, China, the Netherlands, New Zealand, Norway, the United Kingdom, and the United States
Harrer et al (2019) [34]	Systematic review of 48 randomized trials	Internet-based psychological interventions	Anxiety, depression, stress, sleep problems, eating disorders, and well‐being	Mixed nonactive (waitlist, placebo) and active (diaries, recommendations for behavior change)	10,583	Up to 29 years; mean 22 years	Australia, Canada, Finland, Germany, Ireland, Norway, Romania, Spain, Sweden, the United Kingdom, and the United States
Garrido et al (2019) [35]	Systematic review of 27 RCTs and 13 NRTs on 32 interventions and meta-analysis of 15 RCTs	Computer, web-based, and smartphone-delivered intervention	Anxiety and depression	Mixed nonactive (waitlist) and active (alternative therapeutic intervention)	16,874	12-25 years	Australia, Canada, Chile, China, Hong Kong, Ireland, Japan, New Zealand, Northern Europe, the United Kingdom, and the United States
Pretorius et al (2019) [36]	Systematic review of 27 qualitative, feasibility, and comparative studies and 1 RCT	Web-based help-seeking interventions	Psychological distress	No control group	Not reported	12-25 years	Australia, Canada, Ireland, the Netherlands, the United Kingdom, and the United States
Ridout et al (2018) [37]	Systematic review of 9 descriptive studies on 5 interventions	Social networking sites	Depression, psychosis, health literacy, social support, and general well-being	No control group	Not reported	Up to 25 years	Australia, China, Hong Kong, and the United States
Podina et al (2016) [38]	Meta-analysis of 8 RCTs	cCBT^d^	Anxiety	Mixed nonactive (waitlist) and active (standard CBT^e^)	404	7-18 years	Australia, Canada, Spain, and the United States
Ebert et al (2015) [39]	Meta-analysis of 13 RCTs	cCBT	Anxiety and depression	Nonactive (no treatment, placebo)	796	Up to 25 years	Australia, the Netherlands, New Zealand, Sweden, the United Kingdom, and the United States
Pennant et al (2015) [40]	Systematic review and meta-analysis of 27 RCTs	cCBT	Anxiety and depression	Mixed nonactive (waitlist, placebo, no intervention) and active (standard CBT)	3389	5-25 years	Australia, China, the Netherlands, New Zealand, Israel, Sweden, the United Kingdom, and the United States
Ye et al (2014) [41]	Meta-analysis of 7 RCTs	cCBT and SMS	Anxiety and depression	Mixed nonactive (waitlist) and active (standard CBT, alternative intervention)	569	7- 25 years	Australia and the United States
Grist et al (2019) [42]	Meta-analysis of 34 RCTs on 29 interventions	cCBT, computer-delivered attention, or cognitive bias modification programs	Anxiety and depression	Mixed nonactive (waitlist, placebo) and active (face-to-face or alternative therapeutic interventions)	3113	Up to 18 years	Australia, Canada, China, Ireland, Israel, the Netherlands, New Zealand, Sweden, Thailand, the United Kingdom, and the United States
Vigerland et al (2016) [43]	Meta-analysis of 24 RCTs	cCBT	Multiple psychiatric and psychosomatic conditions	Mixed nonactive (waitlist) and active (standard CBT, alternative intervention)	1882	Up to 18 years	Australia, Canada, Germany, the Netherlands, Sweden, and the United States
Clarke et al (2015) [44]	Systematic review of 14 RCTs and 14 NRSs on 21 interventions	Mixed web-based interventions and more than half (8/15) cCBT	Mental health promotion and prevention	Mixed nonactive (waitlist, placebo, no intervention) and active (limited intervention)	10,779	12-25 years	Australia, Canada, China, Germany, Ireland, Israel, the Netherlands, Norway, and the United States

^a^RCT: randomized controlled trial.

^b^NRS: nonrandomized study.

^c^NRT: nonrandomized trial.

^d^cCBT: computerized cognitive behavioral therapy.

^e^CBT: cognitive behavioral therapy.

### Effectiveness Across Clinical Symptom Targets

In terms of clinical outcomes, most systematic reviews and meta-analyses included in this review focused on anxiety (n=4), depression (n=3), anxiety and depression together (n=11), or anxiety and depression with stress (n=3). To a lesser degree, analyses focused on general well-being (n=4). In addition, eating disorders (n=2), psychosis (n=2), attention-deficit/hyperactivity disorder (ADHD; n=1), autism spectrum disorder (n=1), sleep problems (n=1), suicide prevention (n=1), obsessive-compulsive disorder (n=1), role functioning (n=1), phobias (n=1), and posttraumatic stress disorder (n=1) were clinical outcomes explored in the reviews.

Evidence on the benefits of digital mental health interventions was found for anxiety, depression, and stress when compared with nonactive controls, defined primarily as groups to which no treatment was provided or on those put on a waitlist for services. However, compared with active controls, defined as those undergoing or receiving some type of treatment, they appear to be similarly effective (Table 2).

A meta-analysis by Harrer et al [34] on web-based interventions mostly delivered through a dedicated website found small effects on depression (Hedges *g*=0.18; 95% CI 0.08-0.27), anxiety (Hedges *g*=0.27; 95% CI 0.13-0.40), and stress (Hedges *g*=0.20; 95% CI 0.02-0.38) compared with nonactive controls consisting of waitlist or placebo control groups.

A meta-analysis by Davies et al [31] on mixed web-based and computer-delivered interventions for depression, anxiety, and stress found a small effect of digital interventions in comparison with active controls that received alternative materials (for anxiety, pooled standardized mean difference [SMD] −0.18; 95% CI −0.98 to 0.62; *P*=.66 and for depression, pooled SMD −0.28; 95% CI −0.75 to −0.20; *P*=.25), whereas a medium effect was found when compared with nonactive controls. When compared with a nonactive control, there was some effect of decreasing anxiety (pooled SMD −0.56; 95% CI −0.77 to −0.35; *P*<.001), depression (pooled SMD −0.43; 95% CI −0.63 to −0.22; *P*<.001), and stress (pooled SMD −0.73; 95% CI −1.27 to −0.19; *P*=.008).

A meta-analysis by Garrido et al [35] that focused on depression found a small pooled effect size of digital mental health interventions in comparison with nonactive controls (Cohen *d*=0.33; 95% CI 0.11-0.55), whereas the pooled effect size of studies comparing an intervention group with active controls, mostly receiving alternative materials, including website content, showed no significant differences (Cohen *d*=0.14; 95% CI −0.04 to 0.31).

A systematic review by Farrer et al [32] exploring 51 digital interventions using different delivery methods addressing mostly depression, anxiety, and stress found that nearly half of the interventions (24/51, 47%) were associated with at least one positive outcome after the intervention compared with the control group (nonactive and attention controls) and nearly one-third of the interventions (15/51, 29%) failed to report a significant effect. For interventions targeting both symptoms of depression and anxiety (n=8), in comparison with mixed control groups (nonactive and active), effect sizes ranged significantly from −0.07 to 3.04 (overall median 0.54; [effect size] targeting depression symptoms=0.48 and targeting anxiety symptoms=0.77). For interventions targeting only anxiety (n=10), effect sizes ranged from 0.07 to 2.66 (median 0.84). However, the authors of these reviews could not calculate effect sizes for almost two-thirds of the interventions (33/51, 64%) because of insufficient or unavailable meta-data across the reviewed studies [32].

Outcomes of interventions for ADHD, autism spectrum disorders, eating disorders, psychosis, and posttraumatic stress were reported in 3 systematic reviews and one meta-analysis [24,32,34]. Hollis et al [24] demonstrated inconsistent results on the effectiveness of digital interventions for ADHD, autism, psychosis, or eating disorders, limited by the small number of studies and the high degree of variability in reliance on evidence-based treatments. Farrer et al [32] demonstrated the effectiveness of virtual reality or video exposure interventions on arachnophobia or acrophobia. In addition, Harrer et al [34] found moderate effects on eating disorder symptoms (Hedges *g*=0.52; 95% CI 0.22-0.83) and role functioning (Hedges *g*=0.41; 95% CI 0.26-0.56) in comparison with active and nonactive controls (predominantly waitlist control) but no effect on general well-being in comparison with placebo intervention (Hedges *g*=0.15; 95% CI −0.20 to 0.50).

**Table 2:** Key findings of the included studies.

Reference	Effectiveness	Contributing factors	Cost-effectiveness	Inclusion of data on low- and middle- income countries	Quality of included studies	Quality of review (AMSTAR^a^)
Barnes et al (2018) [28]	Although early findings suggest that therapeutic games have the potential to lead to clinically measurable reductions in symptoms in adolescents with anxiety, evidence on the effectiveness is extremely limited. On the basis of 2 RCTs^b^ included in this review, no difference in anxiety outcomes is found between the intervention and control groups (alternative nontherapeutic videogame).	Not discussed	Not discussed	Limited (China and Hong Kong)	Mean rating of 75% using mixed methods appraisal tool. Only 2 RCTs included in the review.	Critically low
Bry et al (2018) [29]	Evidence-based treatment content within consumer smartphone apps marketed for child and adolescent anxiety is scant, and only a few comprehensive anxiety self-management apps are identified. Half of the sampled apps for anxiety include any evidence-based treatment component, and 23% included two or more evidence-based components.	Not discussed	Low cost but effectiveness unknown	No	N/A^c^	N/A
Grist et al (2017) [30]	Authors conclude that there is currently no evidence to support the effectiveness of apps for adolescents with mental health problems. In 2 RCTs on mobile app for depression, anxiety, and stress, no significant effect is found between intervention (app with self-monitoring) and control (no self-monitoring) groups. Acceptability is generally rated average to high, with adherence ranging from 65% to 83%.	Specific factors: privacy, safety, discretion, and data security; credibility of design and visual appearance; engaging and interactive content; concise, interesting, and trustworthy information; reminders to use; and personalization allowed	Not discussed	No	Issues with quality, including small sample size. Only 2 small RCTs included in the review, both without adequate control group.	Critically low
Hollis et al (2017) [24]	cCBT^d^ provides clinical benefits for depression and anxiety when compared with inactive control (waitlist). The benefits for attention deficit hyperactivity disorder and autism are inconsistent, for psychosis are unknown, and eating disorders are no better than waitlist control in regard to symptomology.	Self-guided cCBT has poor uptake and adherence. Human involvement is positively associated with adherence. Adolescents and young people prefer face-to-face over web-based interventions. Specific factors: privacy, safety, discretion, and anonymity; providing concise, interesting, and trustworthy information; and ability to complete interventions on own terms and pace.	Authors note a considerable lack of evidence	Limited (China)	Most studies (18/21) rated as moderate quality, 2 rated as low quality, and 1 rated as high quality using AMSTAR. Methodological issues and high level of heterogeneity in the included studies.	Critically low
Davies et al (2014) [31]	Web-based and computer-delivered interventions are found effective in improving students’ depression (pooled SMD^e^ −0.43; 95% CI −0.63 to −0.22; *P*<.001), anxiety (pooled SMD −0.56; 95% CI −0.77 to −0.35; *P*<.001), and stress (pooled SMD −0.73; 95% CI −1.27 to −0.19; *P*=.008) outcomes when compared with inactive controls (no treatment, waitlist). When compared with active controls (alternative materials), no benefits are found for depression, anxiety, and stress.	Not discussed	Not discussed	No	A moderate risk of bias. Quality issues with reporting of methodology, data, and outcome measures. Only 3 studies with active control, with reported skewed data. Heterogeneity of interventions.	Moderate
Farrer et al (2013) [32]	Approximately half (24/51) of the technology-based mental health interventions targeting tertiary students with anxiety or depression are associated with at least one significant positive outcome, and approximately one-third (15/51) fail to find a significant effect. Effect size for interventions targeting symptoms of depression and anxiety range from −0.07 to 3.04 (median 0.54; depression=0.48; anxiety=0.77). Effect size for interventions targeting symptoms of anxiety range from 0.07 to 2.66 (median 0.84). cCBT was the most deployed therapy in 25 of 51 of the interventions.	Not discussed	Included studies do not report cost-effectiveness	Limited (China)	Mean rating 4.42 out of 9 using Cochrane Effective Practice and Organisation of Care Group. Methodological issues with reporting on randomization, intended outcomes, and heterogeneity of interventions. Insufficient data in more than half of the studies (14/27) to calculate effect sizes.	Low
Valimaki et al (2017) [33]	Web-based mental health interventions yield statistically significant effect on depressive (*P*=.02; median 1.68; 95% CI 3.11 to 0.25) and anxiety symptoms (*P*<.001; median 1.47; 95% CI 2.36 to 0.59) when compared with control group (type not specified), but not on stress (*P*=.14; median 1.06; 95% CI 2.44 to 0.33). After 6 months of intervention, significant improvement is found on depressive symptoms (*P*=.01; median 1.78; 95% CI 3.20 to 0.37), on anxiety symptoms (*P*<.001; median 1.47; 95% CI 2.36 to 0.59), and on moods and feelings (*P*=.04; median 5.55; 95% CI 10.88 to 0.22). Dropout of those in intervention groups was higher than those in control groups.	Interventions with human elements, such as face-to-face guidance or telephone follow-ups, are associated with adherence and effect.	Included studies do not assess costs. Authors note a considerable lack of evidence	Limited (China)	Some risk of bias using Review Manager. Issues include biases related to attrition rates, selective reporting, and small sample sizes. Mixed control groups.	High
Harrer et al (2019) [34]	Internet interventions for university students’ mental health have a small effect on anxiety (Hedges *g*=0.27; 95% CI 0.13 to 0.40), depression (Hedges *g*=0.18; 95% CI 0.08 to 0.27), and stress (Hedges *g*=0.20; 95% CI 0.02 to 0.38) when compared with nonactive controls. Moderate effects were found on eating disorder symptoms (Hedges *g*=0.52; 95% CI 0.22 to 0.83) and role functioning (Hedges *g*=0.41; 95% CI 0.26 to 0.56). Effects on well‐being are nonsignificant (Hedges *g*=0.15; 95% CI −0.20 to 0.50).	Guidance does not significantly affect intervention efficacy (*P*≥.05).	Not discussed	Limited (Romania)	Half of the studies with high risk of bias. Moderate to substantial level of heterogeneity and selective reporting.	Low
Garrido et al (2019) [35]	Digital interventions work better than no intervention (Cohen *d*=0.33; 95% CI 0.11 to 0.55) but not better than active alternatives (alternative web-based materials; Cohen *d*=0.14; 95% CI −0.04 to 0.31) in improving depression in young people, when results of different studies are pooled together. Most interventions were based on CBT^f^. Authors conclude that interventions may be clinically significant only if supervised. Engagement and adherence rates are low.	Interventions with supervision have a higher pooled effect size than those without supervision (studies with no intervention controls: Cohen *d*=0.52; 95% CI 0.23 to 0.80 and studies with active controls: Cohen *d*=0.49; 95% CI −0.11 to 1.01). Specific factors: credibility of design and visual appearance; concise, interesting, and trustworthy resources; engaging and interactive tools and content; esthetically attractive; relatable situations, characters, or avatars; and reflect local and cultural differences and needs. Technical glitches as a barrier to complete interventions.	Not discussed	Limited (China, Hong Kong, and Chile)	On the basis of Joanna Brigg Institute appraisal tool and CONSORT (Consolidated Standards of Reporting Trials), 32 of 41 studies with high or unclear overall bias and 9 of 41 with low overall bias.	Low
Pretorius et al (2019) [36]	N/A	Young people value web-based services because of anonymity, accessibility, self-reliance, and ease of use. Theoretical frameworks, including self-determination theory and help-seeking model, should be deployed in research. Specific factors: anonymity, privacy, safety, and discretion; site moderation by professionals; credibility of design and visual appearance; concise, interesting, and trustworthy information; esthetically attractive; flexibility, self-reliance, and control; and 24-h availability.	Not discussed	No	Moderate to strong using Critical Appraisal Skills Program. Heterogeneity of interventions. Only 1 RCT included in the review.	Critically low
Ridout et al (2018) [37]	Social networking sites targeting mental health have significant improvement in mental health knowledge and a number of depressive symptoms in young people, but no improvement in anxiety or psychosis symptoms. The results are not compared with a control group.	Young people value involvement of professionals and peers in social networking sites.	Authors conclude that web-based interventions are cost-effective but provide no evidence	Limited (China and Hong Kong)	No quality assessment performed. On the basis of descriptive studies, no RCTs included in the review.	Critically low
Podina et al (2016) [38]	cCBT is as effective as standard CBT (Hedges *g*=0.295) and more effective than waitlist (Hedges *g*=1.410) in reducing anxiety symptoms in anxious children and adolescents.	Not discussed	Not discussed	No	No quality assessment performed. No publication bias found. Only 8 RCTs included in the review.	Critically low
Ebert et al (2015) [39]	cCBT for youth is associated with significant moderate to large effects on symptoms of anxiety (Hedges *g*=0.68; 95% CI 0.45 to 0.92; *P*<.001) and depression (Hedges *g*=0.68; 95% CI 0.45 to 0.92; *P*<.001) in comparison with nonactive controls. Effect size on symptoms of anxiety or depression for cCBT was similar to face-to-face CBT (Hedges *g*=0.72 vs Hedges *g*=0.66) and higher than face-to-face CBT targeting depression (Hedges *g*=0.35).	No association between parental involvement and better outcomes (without parental involvement: Hedges *g*=0.83; 95% CI 0.53 to 1.13; *P*<.001; NNT^g^=2.26 and with parental involvement: Hedges *g*=0.64; 95% CI 0.40 to 0.88; *P*<.001; NNT=2.86)	Not discussed	No	Low risk of bias overall. Low heterogeneity	Low
Pennant et al (2015) [40]	cCBT has positive effects for symptoms of anxiety (SMD 0.77; 95% CI 1.45 to 0.09; n=6; number of participants=220) and depression (SMD 0.62; 95% CI 1.13 to 0.11; n=7; number of participants=279) for young people with risk of diagnosed anxiety and depression disorders. cCBT has lower effect size on anxiety (SMD 0.15; 95% CI 0.26 to 0.03; number of participants=1273) and depression (SMD 0.15; 95% CI 0.26 to 0.03; number of participants=1280) in the general population. Evidence for interventions other than cCBT is sparse and inconclusive.	Not discussed	Not discussed	Limited (China)	On the basis of Grading of Recommendations, Assessment, Development and Evaluation evidence quality review, most studies rated from very low (1/17) to low (11/17) to moderate (5/17). Heterogeneity associated with number of outcomes.	Critically low
Ye et al (2014) [41]	When compared with inactive controls, cCBT is effective in reducing anxiety symptoms (SMD −0.52; 95% CI −0.90 to −0.14) but not depression (SMD −0.16; 95% CI −0.44 to 0.12). No significant difference is found when compared with standard face-to-face CBT, suggesting it is as effective.	Not discussed	Included studies do not report on cost-effectiveness	No	On the basis of Quality Assessment Tool for Quantitative Studies, studies rated high (3/7) and moderate (4/7) quality. Only 7 RCTs included in the review.	Critically low
Grist et al (2019) [42]	A small effect (n=8; Hedges *g*=0.41; 95% CI 0.08 to 0.73; *P*<.01) is found in technology-delivered mental health interventions related to attention bias modification when compared with waitlist controls. Although cCBT interventions yield a medium effect size, attention bias modification programs yield a small effect size, and cognitive bias modification programs yield no effect size.	Therapist support (Cochran Q=27.28; *P*<.001) as well as parental involvement (Cochran Q=24.43; *P*<.001) have a significant effect on effectiveness of and adherence to an intervention. Therapist involvement yields a higher effect size (n=9; Hedges *g*=0.87; 95% CI 0.68 to 1.06; *P*<.001) than predominantly or purely self-administered interventions.	Authors note a considerable lack of evidence	Limited (China)	Most studies rated as low quality and unclear risk using Cochrane Risk of Bias Tool. Most studies (29/34) conducted by program developer. Methodological limitations, small sample size, and nonblinding participants.	Low
Vigerland et al (2016) [43]	cCBT yields moderate effects when compared with waitlist controls (Hedges *g*=0.62; 95% CI 0.41 to 0.84).	Not discussed	Authors note a considerable lack of evidence	No	Quality varied largely across the studies; Moncrieff mean 30.2 of 46. Heterogeneity of measures included.	Low
Clarke et al (2015) [44]	There is some evidence that skills-based interventions presented in a module-based format can have a significant impact on promoting adolescent mental health and that cCBT has significant positive effects on adolescents’ anxiety and depression symptoms; however, research is limited. Improvements of symptoms are maintained at 6 and 12 months.	Face-to-face and web-based support are associated with improved program completion and outcomes.	Not discussed	Limited (China)	On the basis of Quality Assessment Tool for Quantitative Studies, quality varied significantly from weak (12/20) to moderate or strong (7/20). Issues include a small number of studies, poor sampling, and heterogeneity across interventions.	Low

^a^AMSTAR: A Measurement Tool to Assess Systematic Reviews.

^b^RCT: randomized controlled trial.

^c^N/A: not applicable. This is a systematic review of apps and not studies, and therefore, quality assessment is not applicable.

^d^cCBT: computerized cognitive behavioral therapy.

^e^SMD: standardized mean difference.

^f^CBT: cognitive behavioral therapy.

^g^NNT: number needed to treat.

In conclusion, converging evidence across reviews suggests that digital health interventions have a small to medium effect when compared with nonactive controls (ie, waitlist or placebo). When compared with active controls, digital health interventions appear to be comparable, although findings varied by targeted set of symptoms, with evidence of effectiveness most apparent for anxiety and depression and to a lesser extent for stress. Inconclusive results across other symptom types were because of the limited number of trials conducted to date.

### Effectiveness of Clinical Interventions

Most systematic reviews and meta-analyses have reported findings across studies that test the effectiveness of the implementation of computerized cognitive behavioral therapy (cCBT) [33,34,38-42,44]. Investigations of digital mental health interventions other than cCBT are rare, and thus, our analysis on the effectiveness of digital clinical interventions across studies focuses exclusively on cCBT.

According to 4 reviews, there is no significant difference in the effectiveness between cCBT delivered through a digital platform and standard face-to-face cognitive behavioral therapy (CBT) [38,39,41]. However, there is some evidence of benefits compared with nonactive controls [31,34,35,39,42].

Ye et al [41] found no statistical difference between internet-based CBT and face-to-face interventions, suggesting that the digital format may retain effectiveness. However, when compared with nonactive controls, cCBT was effective in reducing anxiety symptoms (SMD −0.52; 95% CI −0.90 to −0.14) but not in reducing depression (SMD 0.16; 95% CI 0.44-0.12) [41]. A meta-analysis by Podina et al [38] found that cCBT was as effective as standard CBT (Hedges *g*=0.295) and more effective than waitlist (Hedges *g*=1.410) in reducing anxiety symptoms. Similarly, Vigerland et al [43] found a moderate effect on social anxiety disorder compared with waitlist controls (Hedges *g*=0.62; 95% CI 0.41-0.84). In 2 separate trials, older participants were found to gain greater clinical benefits compared with younger participants (slope=0.514) [24,38].

Ebert et al [39] found that the overall mean effect size of cCBT on symptoms of anxiety or depression was Hedges *g*=0.72 (95% CI 0.55-0.90) at posttest after controlling the baseline levels. This effect is similar to the effect of traditional CBT for anxiety (0.66) and higher than that of CBT for the treatment of depression in youth (0.35). When compared with a nonactive control, cCBT was effective in targeting anxiety (Hedges *g*=0.68; 95% CI 0.45-0.92; *P*<.001) and depression (Hedges *g*=0.76; 95% CI 0.41-0.12; *P*<.001).

With regard to studies with mixed comparison groups (active and nonactive), Harrer et al [34] found cCBT interventions more effective than others (eg, relationship skills training and emotional disclosure) for some conditions (depression: Hedges *g*=0.28; 95% CI 0.15-0.40 vs Hedges *g*=0.04; 95% CI −0.23 to 0.30; number needed to treat [NNT]=6.41 vs 4.4.5 and anxiety: Hedges *g*=0.36; 95% CI 0.23-0.50 vs Hedges *g*=−0.06; 95% CI −0.46 to 0.35; NNT: 5 vs 29.41). Similarly, Clarke et al [44] found that module-based cCBT showed significant positive effects in reducing depression and anxiety, thoughts of self-harm, and hopelessness and in improving sense of control.

Pennant et al [40] demonstrated greater effects when cCBT is targeted to young people assessed at risk of anxiety or depression, in comparison with the general population of young people. Among young people with elevated depression or anxiety symptom scores, cCBT had positive effects on anxiety (SMD 0.77; 95% CI 1.45-0.09; number of studies, n=6; number of participants=220) and depression (SMD 0.62; 95% CI 1.13-0.11; n=7; number of participants=279), whereas in the general population of young people, effect sizes were smaller (anxiety: SMD 0.15; 95% CI 0.26-0.03; number of participants=1273 and depression: SMD 0.15; 95% CI 0.26-0.03; number of participants=1280) [33]. Similar findings were also found in 2 other systematic reviews [34,44].

With regard to non-cCBT interventions, a small effect size of attention bias modification programs for anxiety and depression was observed (n=8; Hedges *g*=0.41; 95% CI 0.08-0.73; *P*<.01), whereas no benefit of cognitive bias modification programs or other interventions over either passive or active control groups (other therapeutically active conditions, attention or placebo training conditions, and waitlist) was observed [42].

### Effectiveness of Digital Platforms

Only 4 systematic reviews have reported findings on digital platforms used to deliver digital mental health services. These included social networking sites [37], mental health apps [18,29], and therapeutic video games [28].

A systematic review by Ridout and Campbell [37] on social networking sites targeting mental health found no evidence of improvement in anxiety or psychosis symptoms in young people, whereas it found improvements in enhancing mental health knowledge and the number of depressive symptoms. Among the sites, the review suggested that the closed Facebook-like moderated online social therapy platforms as well as the YBMen project that used Facebook was effective, although there was no evidence of the effectiveness of other social networking platforms (the MindMax and Ching Story) included in the review [37]. In another systematic review, Grist et al [18] found no evidence to support the effectiveness of apps designed for adolescents with mental health conditions.

One reason for the lack of effectiveness across specific platforms may be attributable to a limited evidence base for many of the interventions available. For example, a review of 121 anxiety apps available in app stores (Google and Apple) by Bry et al [29] found that only a limited number of these apps were evidence based. Only one-sixth of the apps included educational information on the definition, symptoms, and treatment of anxiety. Half had at least one evidence-based treatment component, and one-fourth had more than one evidence-based treatment component, such as exposure therapy; thought challenging or cognitive restructuring; or self-monitoring of one’s thoughts, emotions, and behaviors. The majority of those that lacked any evidence-based components were mostly distraction tools, such as games, coloring activities, or other audio or visual activities, and more than half included relaxation exercises, which are currently rarely considered therapeutic for anxiety [29]. Evidence on the effectiveness of therapeutic video games was limited and mixed, as confirmed by Barnes and Prescott [28].

Irrespective of their effectiveness or link with evidence-based approaches, young people generally perceive their engagement with these platforms to range from neutral to helpful. Overall, a systematic narrative review of Pretorius et al [36] reported that young people’s perception of the helpfulness of web-based resources ranged across the studies—from 80% of participants in a study indicating that speaking on the web had helped, to 40% reporting in another study that web-based resources had helped a little, to 59% reporting in a third study that web-based resources did not make things better or worse.

### Factors Associated With Effectiveness and Adherence

Several systematic reviews and meta-analyses demonstrated that digital mental health interventions with an in-person element (ie, therapist, parent, and peer) were more effective than those that were fully automatized or self-administered.

In another systematic review, Grist et al [42] found a significant effect of therapist support (Cochran Q=27.28; *P*<.001) and parental involvement (Cochran Q=24.43; *P*<.001). In their analysis, the involvement of a therapist yielded higher effect sizes (n=9; Hedges *g*=0.87; 95% CI 0.68-1.06; *P*<.001) than predominantly self-administered (Hedges *g*=0.81; 95% CI −0.68 to 2.31; *P*=.29) or purely self-administered interventions (Hedges *g*=0.24; 95% CI 0.10-0.38; *P*<.001). Similar findings were also reported by Hollis et al [24].

Garrido et al [35] reported higher pooled effect sizes of digital mental health interventions for depression with supervision than those without supervision (studies with no intervention controls: Cohen *d*=0.52; 95% CI 0.23-0.80 and studies with active controls: Cohen *d*=0.49; 95% CI −0.11 to 1.01). In a systematic review and meta-analysis, Valimaki et al [33] found that web-based interventions with a human element, including face-to-face guidance, monitoring of engagement, or follow-up telephone calls by teachers and health professionals, were more effective than those without a human element.

Grist et al [42] demonstrated a significant difference in effect sizes (Cochran Q=9.37; *P*=.002) between trials with ongoing psychological or pharmacological treatment (Hedges *g*=0.90, 95% CI 0.68-1.11; *P*<.001) and trials without ongoing treatment (Hedges *g*=0.42, 95% CI 0.20-0.63).

In contrast, Harrer et al [34] did not find supervision significantly affecting intervention efficacy; however, this may be because of the multiplicity of the types of interventions included in the review or the older target population (university students). In addition, Ebert et al [39] found no association between parental involvement and better treatment outcomes of cCBT for anxiety or depression in youth (without parental involvement: Hedges *g*=0.83, 95% CI 0.53-1.13; *P*<.001; NNT=2.26 and with parental involvement: Hedges *g*=0.64, 95% CI 0.40-0.88; *P*<.001; NNT=2.86).

An in-person element was also associated with adherence and lower dropout rates. Clarke et al [44] suggested that face-to-face or web-based support in web-based interventions was associated with better completion and outcomes. Similarly, Hollis et al [24] reported that human involvement is positively associated with adherence; however, they note that the evidence is scant.

Human contact in digital mental health interventions was also considered useful and valuable by adolescents and young people themselves, in particular, contact with professionals as well as peers with similar experiences and mental health issues [35,37]. Pretorius et al [36] found that young people valued web-based services run by mental health professionals and the opportunity to connect to peers, with 84% of participants reporting that human contact within a web-based mental health resource is important. In addition, in a systematic review by Ridout and Campbell [37], the involvement of professionals and peers in social networking sites was valued by site users.

Hollis et al [24] reported that adolescents and young people prefer face-to-face mental health interventions over digital interventions. In the Australian sample, two-thirds (59%) of young people strongly preferred face-to-face treatment, with only 16% preferring on the web, and in the United Kingdom, half were not interested in cCBT, with preference for face-to-face treatment.

There was some indication that interventions implemented in the school setting were associated with improvements in adolescent mental health knowledge, support seeking, and well-being [44]. School- and web-based interventions were also associated with greater adherence [35,44], and interventions that adolescents and young people completed in their own time were associated with low completion rates and adherence [35].

### Design Elements

Acceptability of interventions was reported to be good [30,37,40,44]. Privacy, safety, and discretion were found to be valuable for adolescents and young people [24,30,36]. Related to the stigma associated with mental health issues, adolescents and young people also valued anonymity [24,36]. In this regard, data security, including password protection, control over privacy settings [30], and site moderation by professionals [36,37], were identified as factors influencing the acceptability of digital mental health interventions.

Other characteristics valued by adolescents and young people included the credibility of design, visual appearance, and information and resources provided [30,35,36]. The tools and content should be engaging and interactive [30,35]; should provide concise, interesting, and trustworthy information [24,30,35,36]; should be esthetically attractive [35,36]; should provide reminders to use [30]; should allow for personalization [30]; should have relatable situations, characters, or avatars [35]; and should reflect local and cultural differences and needs, particularly in terms of minority groups and migrants for social integration. Garrido et al [35] reported that technical glitches were a barrier to complete interventions.

Flexibility, self-reliance, and control were also cited in the reviews as influencing acceptability [24,36]. Adolescents and young people valued in digital mental health interventions the ability to complete interventions on their own terms and pace [24]. According to Pretorius et al [36], 24-hour availability is an important factor, as help-seeking takes place mostly after 11 PM.

### Sustainability, Completion, and Adherence

Most studies included in this review reported only short-term effects on adolescents’ mental health. Evidence of long-term effects is limited [24,33,38-41,44]. Only one meta-analysis by Valimaki et al [33] with a focus on depression, anxiety, and stress examined the long-term effects of digital health interventions. The study found a statistically significant improvement at the end of the intervention on depressive symptoms (*P*=.02; median 1.68, 95% CI 3.11-0.25) and after 6 months (*P*=.01; median 1.78, 95% CI 3.20-0.37). The study also found evidence of long-term improvement at 6 months in anxiety symptoms (*P*<.001; median 1.47, 95% CI 2.36-0.59) and moods and feelings (*P*=.04; median 5.55, 95% CI 10.88-0.22), but there was no difference in stress scores.

In terms of cCBT, in line with the standard CBT, effects were higher for interventions of moderate length (1-2 months), for example, on depression at 4-8 weeks (Hedges *g*=0.31, 95% CI 0.13-0.49; NNT=5.75) compared with shorter (Hedges *g*=0.09, 95% CI −0.02 to 0.21; NNT=20) or longer (Hedges *g*=0.13, 95% CI −0.43 to 0.69; NNT=13.51) programs (*P*=.03), according to Harrer et al [34]. Although follow-up assessments were rarely reported in studies, Clarke et al [44] also found that improvements after cCBT were maintained at 6 and 12 months.

In addition to limited evidence of the long-term effects of digital mental health interventions, Hollis et al [24] found limited evidence of a dose-response (ie, how much of the intervention is needed to produce beneficial outcomes).

Overall, dropout was found to be high in the systematic reviews and meta-analyses of studies on digital mental health interventions. Completion rates ranged greatly from 10% to 94% in a study by Valimaki et al [33] and from 65% to 83% among app users in a study by Grist et al [30], and completion rates were approximately half on average in a study by Clarke et al [44]. However, data on dropout and adherence were generally considered weak in the original review samples, with only a limited number of studies reporting data on adherence [24,30,33,35,36,44].

Gender was considered as a predictor of adherence. According to Garrido et al [35], females were more likely to complete the intervention than males [35]. In addition, mental health status was associated with completion, and higher completion was predicted for adolescents and young people with higher depression scores at the baseline [35,44], a longer history of mood disorders, or low anxiety scores at pretest [36]. Furthermore, according to Pretorius et al [36], high levels of psychological distress were associated with help-seeking on the web.

### Cost-Effectiveness

Data on cost-effectiveness were not reported in any of the systematic reviews and meta-analyses in our sample, and there was no indication of research and development costs. A total of 5 systematic reviews noted that despite being widely considered low cost, for example, because of reduced time and personnel expenses [43], there is still a lack of data on the cost-effectiveness and economic benefits of digital mental health interventions [24,29,32,33,42].

### Generalizability of Findings

None of the studies reported on the socioeconomic background or other characteristics of the target populations. Most studies were conducted in high-income countries across Europe (n=71) and in the United States (n=21), Australia (n=21), Canada (n=13), and New Zealand (n=9). In terms of low- and middle-income economies, interventions were reported only from 4 countries, with most studies conducted in China (n=9), including Hong Kong, and, to a lesser extent, in Chile (n=2), Egypt (n=1), and Thailand (n=1). Given the homogeneity of the country contexts and lack of analysis of the characteristics of the target population, the generalizability of the findings is limited beyond adolescents and young people in high-income country settings.

## Discussion

### Principal Findings

We explored 18 reviews and meta-analyses on the effectiveness of digital mental health interventions for adolescents and young people. On the basis of this systematic overview, we found evidence on the effectiveness of cCBT on anxiety and depression, whereas the effectiveness of other digital mental health interventions, including therapeutic video games, mobile apps, or social networking sites, remains inconclusive. The effects vary based on a targeted set of symptoms, with evidence of effectiveness found on anxiety; depression; and, to a lesser extent, stress, and based on age, with older participants gaining greater benefits compared with younger adolescent participants.

Digital interventions that deploy evidence-based treatment such as cCBT are generally comparable with face-to-face care. Importantly, in-person elements (eg, professional, peer, or parent engagement) were found to strengthen the effectiveness of digital interventions. In addition, digital interventions improved outcomes relative to waitlist controls, suggesting that they may have additional benefits for supporting adolescents and young people in cases where access to care is limited or wait times to access are long.

Furthermore, although young people report a range of neutral to positive attitudes about the helpfulness of digital platforms for mental health support, few studies have tracked the long-term outcomes of digital mental health interventions. Although acceptability is considered good, dropout is common, and adherence is relatively weak if not boosted by in-person elements. Very little is known about cost-effectiveness, with no systematic reviews or meta-analyses reporting on cost-effectiveness. Finally, given that the vast majority of interventions are implemented in high-income countries, very little is known about the generalizability of the findings to low- and middle-income countries and to a range of adolescents and young people with different socioeconomic, cultural, racial, or other backgrounds.

Despite some converging evidence across meta-analyses and reviews, research in this area appears to have consistently low quality and rigor as per assessment using the AMSTAR 2 criteria. The primary constraints for this were that the articles analyzed reported many limitations in their samples. These included a small number of studies meeting the inclusion criteria [28,30,31,38,39,41,44], weak quality of studies [32,34,40,41,43,44], and the heterogeneity across the interventions in terms of content and delivery [24,31,34,36,39-41,44]. Furthermore, study participants were often recruited by self-selection [30,37,44], sample sizes were small [24,30,32], and blinding was limited [24,30,35]. Notably, one systematic review by Grist et al [30] also pointed out that almost all studies were either undertaken or supported by the program developer, which may greatly affect the study design and interpretation of the findings.

### Comparison With Previous Work

With the growing application of digital technologies in public health, digital health interventions are perceived to increase access to health services and information, self-care, and empowerment and reduce the cost and burden on health systems [45]. In this context, as *digital natives*, adolescents and young people are considered as early adopters of technology [46], with the potential to benefit from digital health technologies, including for mental health.

Although there is an increasing body of research on the effectiveness of digital mental health technologies targeting adolescents and young people, most focus on evaluating cCBT. In line with our findings, cCBT for addressing anxiety and depression in adolescents and young people has been found to be effective, including in school-based prevention and early identification studies and in family-based studies [47]. The effectiveness of cCBT in the adult population has also been established [48,49]. Given that face-to-face CBT is widely used as a treatment for depressive symptoms and disorders in this age group [50], with evidence of its effectiveness found in a number of systematic reviews [51-53], it is plausible that it also works in a standardized digital format.

Beyond the cCBT, evidence on the effectiveness of other digital mental health interventions, including therapeutic video games, mobile apps, and social networking sites, was extremely limited. Although these may have the potential to engage adolescents and young people and thus support traditional face-to-face treatment [54] and although social network sites, including gaming elements, are found to be promising in promoting changes in health-related behaviors [55,56], the quality of content and expected outcomes vary [57].

Similar to our findings, studies have reported low adherence and high dropout rates in adolescents and young people using digital mental health interventions [58-60], although there are also contrasting data with high levels of acceptability and usability [47], including from low- and middle-income countries [61-63]. However, the contrasting data are mainly reported in feasibility studies, based on adolescents without mental health conditions, and thus, the data may not be applicable for adolescents and young people with mental health issues.

Furthermore, to some extent, the cost-effectiveness of digital health interventions has been studied in the general population and other areas of health, including the management of cardiovascular diseases [64] and insomnia [65]. However, there is a lack of assessment of cost-effectiveness in digital mental health interventions overall and for adolescents and young people in particular. This may be because of methodological limitations related to a number of studies, including heterogeneity of interventions and outcomes that hinder the overall assessment of effectiveness.

Finally, despite an increasing share of young population and users of digital technology in low- and middle-income countries, very little research has been conducted in these settings [61,62]. In line with our findings, the generalizability to low- and middle-income countries [47,66] as well as adolescents and young people with different backgrounds [47] is noted by previous research. However, good-quality research on cost-effectiveness and generalizability is critical when scaling up these interventions in settings with already limited resources for health care, including mental health services.

### Limitations

Although this overview of meta-analyses and systematic reviews provides a broad assessment of the results and quality of digital mental health intervention research focused on adolescents and young people, several limitations are evident. In this overview, we have provided a higher-level synthesis of previous systematic reviews in this area, covering a range of digital health interventions and expected health outcomes. Although this is a critical step in assessing the value of digital interventions overall, it introduces some challenges for interpretation (eg, variation in study settings, methods, and comparators, with inconsistencies in reporting within and across the reviews, including the level of description of primary studies and the findings). However, these inconsistencies highlight an important need for more systematic approaches to testing and reporting on effectiveness across studies. Inclusion criteria for some of the studies reviewed here may have resulted in overlap of primary studies between the reviews. In addition, as the field of digital interventions is fast-moving, many of the interventions tested may now be outdated or defunct. However, cross-study heterogeneity is why this review is needed to identify converging effects that emerge, despite variation in specific tests across studies and reviews.

Finally, we included only published peer-reviewed systematic reviews and meta-analyses in the English language. Inclusion of randomized control trials and other original research, including in other languages, may have yielded more studies focused on low- and middle-income countries.

Despite these limitations, the present overview provides a broad picture of the converging evidence supporting the promise of digital mental health interventions in adolescents and young people and highlights a critical need for the field to increase the number of high-quality effectiveness trials to ensure that the interest and enthusiasm in these approaches do not outpace their results.

### Conclusions

This overview of meta-analyses and systematic reviews suggests that digital mental health interventions for adolescents and young people have modest positive effects, especially when relying on evidence-based treatment content or in-person elements that boost engagement. Their potential for settings with limited resources for health and cost savings compared with traditional treatment remains understudied. Therefore, when developing, investing in, and delivering digital mental health programs for adolescents and young people, we need to better consider what types of services are meaningful to be provided through a digital platform (ie, cCBT that deploys the same techniques as face-to-face therapy and is typically delivered by a professional), for what outcomes (eg, self-reported vs diagnosed and mild vs severe symptoms), what type of services adolescents and young people themselves prefer (standard vs digital), and to what extent these are cost-saving and clinically effective across a variety of settings with different resources (ie, in high- vs low-resource settings).

## Abbreviations

ADHD: attention-deficit/hyperactivity disorder
AMSTAR 2: A Measurement Tool to Assess Systematic Reviews
CBT: cognitive behavioral therapy
cCBT: computerized cognitive behavioral therapy
NNT: number needed to treat
SMD: standardized mean difference
